# Outreach strategies to promote HIV testing and linkage-to-care focusing on a young sexual and gender-diverse population in Bangkok, Thailand

**DOI:** 10.1371/journal.pone.0296130

**Published:** 2024-01-11

**Authors:** Paponsan Chiaprasert, Rangsima Lolekha, Supattra Rungmaitree, Alan Maleesatharn, Chuenkamol Sethaputra, Yuitiang Durier, Pornchai Srisoonthonthai, Wachara Pumpradit, Sanny Chen Northbrook, Peerawong Weerarak, Kulkanya Chokephaibulkit

**Affiliations:** 1 Division of Infectious Disease, Department of Pediatrics, Faculty of Medicine Siriraj Hospital, Mahidol University, Bangkok, Thailand; 2 Division of Global HIV and TB, U.S. Centers for Disease Control and Prevention Thailand Office, Nonthaburi, Thailand; 3 Path2Health Foundation, Bangkok, Thailand; 4 Bangkok Health Hub, Bangkok, Thailand; 5 Division of Preventive Medicine, Department of Medicine, Faculty of Medicine Siriraj Hospital, Mahidol University, Bangkok, Thailand; 6 Siriraj Institute of Clinical Research, Faculty of Medicine Siriraj Hospital, Mahidol University, Bangkok, Thailand; University of Zimbabwe Faculty of Medicine: University of Zimbabwe College of Health Sciences, ZIMBABWE

## Abstract

**Introduction:**

Human Immunodeficiency Virus (HIV) prevalence among young gender-diverse (a wide range of gender identities for people whose gender identity is different from the sex that they were assigned at birth) individuals is high but testing coverage among this key population remains low. We aim to evaluate strategies for outreach, HIV testing, and linkage to proper management in young men-who-have-had-sex-with-men (MSM, homosexual male) and transgender women (TGW) in Bangkok, Thailand.

**Methods:**

The “YM2M outreach program” consisted of two strategies: 1) online platforms (OP) and 2) physical outreach activities (POA). Participant questionnaires were completed on a voluntary basis during outreach activities during 2018–2021. Demographic and behavioral characteristics were assessed for association with HIV positivity.

**Results:**

A total of 3,972 homosexual male and TGW participated in the YM2M program: 2,973 by OP and 999 by POA. Of 2,230 participants who reported gender identity, 603/1,392 (43.3%) of OP and 252/985 (25.6%) of POA were gender diverse. Of 631 (21.2%) participants in OP and 970 (97.1%) in POA who underwent testing, 286 (45.3%) in OP and 41 (4.2%) in POA were HIV-positive. The venue reporting highest HIV yield was the Mor-Lam (11.5%). Among those with an HIV-positive test, 175 (61.2%) from OP and 23 (51.1%) from POA were successfully linked to HIV care. The independent factors associated with HIV positive in OP were being youth (adjusted odd ratio (aOR), 0.37; 95%CI 0.16–0.81; P = 0.01) and suspected or confirmed STI (aOR 15.39; 95%CI 7.17–33.03, P<0.01); while those in in POA at Mor-Lam were being gender diverse (aOR, 8.43; 95%CI 1.94–36.62; P<0.01) and reactive syphilis test (aOR, 5.40;95%CI 2.45–11.88; P<0.01). Linkage to pre-exposure prophylaxis (PrEP) among HIV-negative participants was low, 4.9% and 2.6% in OP and POA participants, respectively.

**Conclusions:**

While uptake of HIV testing was higher in POA while OP was more effective in identifying undiagnosed people living with HIV/AIDS and linking them to care. Neither strategy was considered effective in linkage to PrEP.

## Introduction

Although the number of new HIV infections in Thailand has decreased by about 56% since 2010 [1], the prevalence among sexual-diverse groups such as men-who-have-had-sex-with-men (MSM, homosexual males) and transgender women (TGW) remains disproportionately high [1, 2]. The highest prevalence of HIV was reported among MSM (7.3%) and TGW (4.2%) compared to other groups in 2020 [3]. Adolescents accounted for about 4% of all people living with HIV and about 11% of new HIV infections [1]. Gender-diverse youth are therefore the most important target groups for strategic outreach to prevent transmission and acquisition of HIV [4, 5].

HIV testing is the key initial step for HIV prevention and treatment services [2, 6]. In 2020, UNAIDS estimated that merely 52.8% of MSM and 68.4% of transgender (TG) population underwent HIV testing and were aware of their status [1]. A study by Khawcharoenporn T. et al. indicated that 34% of 358 MSM in a gay sauna in Thailand were unaware of their HIV status; 24% of those men were 15–24 years of age and first-time testers [7, 8]. Due to the low rates of HIV testing among this population, interventions that render testing more accessible are critical to the implementation of a successful HIV prevention strategy.

The strategies to reach populations of focus in different settings need to be tailored based on culture and societal environment. Outreach HIV testing activities would be effective and convenient if implemented at established gathering venues popular with populations of focus such as pubs, bars and concerts. Implementing outreach activities in schools could potentially reach youth at higher risk of HIV and sexually transmitted infections (STI) and provide appropriate education and raise HIV awareness [9]. As internet users aged 16 to 64 years in Thailand spend an average of 3 hours daily on social media [10], this platform provides another opportunity for reaching and recruiting youth at higher risk of HIV and STI for HIV Testing and Counseling. Peer-to-peer word of mouth can also be effective by social media [11]. Additionally, literature regarding outreach strategies in resource-limited settings is sparse.

**Fig 1:**
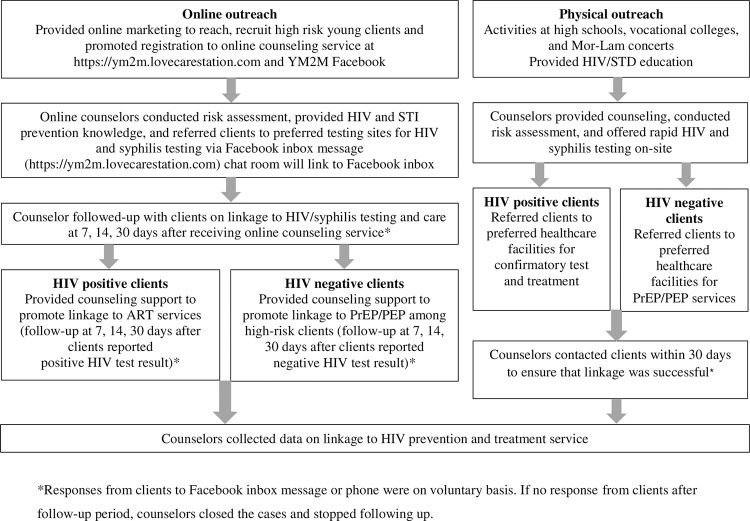
Recruitment algorithm for YM2M outreach program: Online Outreach Platform (OP) from September 2018 to July 2021 and Physical Outreach Activities (POA) from July 2018 to February 2020, Bangkok, Thailand. *Responses from clients to Facebook inbox message or phone were on voluntary basis. If no response from clients after follow-up period, counselors closed the cases and stopped following up.

The “YM2M program” is an outreach program developed to promote, reach, recruit, test, and link young MSM and TGW to HIV and STI services adapted to the Thai context. The program was an initiative from the Division of AIDS and STI of the Ministry of Public Health (MOPH), Siriraj Hospital, PATH2HEALTH Foundation (P2H), Bangkok Health Hub (BHH) private clinic, Department of Medical Services and the Department of Health (DOH) under the Bangkok Metropolitan Administration (BMA), The United Nations Children’s Fund of Thailand (UNICEF Thailand), and the U.S. Centers for Disease Control and Prevention (CDC), Thailand office. The YM2M program consists of two outreach strategies: 1) online platforms (OP) using web-based education and social media communication designed to attract young MSM and TGW and 2) physical outreach activities (POA) to recruit and test young people at risk for HIV particularly MSM and TGW at selected venues, including high schools and vocational colleges. Youth at higher risk of HIV and STI were linked to HIV prevention services and youth with HIV were linked to treatment services (Fig 1).

This study aims to evaluate the efficacy of the outreach strategies in the YM2M program to link youth at higher risk of HIV and STI to prevention services and HIV-positive youth to treatment services. Lessons learned from the YM2M program will inform effective online and outreach strategies to reach young MSM and TGW for wider scale implementation.

## Materials and methods

### Study designs and populations

This is a retrospective study of participants reached by the YM2M program in 2018 before the emergence of COVID-19. Youth is defined as participants who were 13 to 24 years of age. Adult group is defined as participants at least 25 years of age. In this study, gender-diverse is defined as participants who self-reported as homosexual males or MSM, TGW or bisexual. While cisgender is defined as a person whose gender identity was the same as their sex assigned at birth. The YM2M program consists of two outreach strategies: OP and POA. OP was conducted from September 2018 through July 2021 while POA was conducted by Siriraj staff and Bangkok Metropolitan Administration Department of Health team from July 2018 through February 2020. During the initial phase in 2018–2019, POA was conducted at 11 high schools and 5 vocational colleges. However, due to the low HIV yield in schools, the strategy shifted in 2019 to focus on MSM and TGW at higher risk of HIV and STI at gathering venues including one sauna place for men and 9 Mor-Lam concerts, a popular Thai folk concert of which the majority of performers are MSM and TGW.

The OP outreach strategy provided web-based demand-created materials for education in reproductive health, sex, and sexually transmitted diseases (https://ym2m.lovecarestation.com, UNICEF-sponsored comprehensive adolescent health care website managed by P2H) and live-online counseling using social media (Facebook) available from 10 am to midnight every day, with links to HIV testing and medical care, as well as referrals to off-line or face-to-face counseling at a health care facility as needed. The online pages and activities were designed to attract young people, particularly young MSM and TGW. The HIV counseling services were free of charge to the participants. All participants completed the registration form voluntarily before initiating the online counseling appointment. Online counselors conducted risk assessment, provided HIV and STI prevention knowledge, introduced HIV testing and pre-exposure prophylaxis (PrEP) services, and offered linkage to HIV testing based on the client’s preference for HIV testing sites. Online counselors followed up with all clients a total of 3 times in a 30-day period using chat texting to inquire about testing status and need for further support or care. For clients receiving HIV testing, the counselor provided additional support to ensure clients were linked to HIV prevention and treatment service. HIV-negative participants with high-risk behaviors were linked to PrEP or post-exposure prophylaxis (PEP) services as needed while HIV-positive participants were encouraged to take antiretroviral therapy (ART) at the same health care facilities based on the client’s health care scheme. The counselor discontinued follow up after 30 days of online service or 3 unsuccessful attempts (i.e., participants did not provide text responses or refused HIV testing within 30 days).

We conducted educative POA in 11 high schools and 5 vocational colleges, all invited based on the high proportion of gender-diverse youth reported by college counselors. POA activities were designed with specificity to each venue’s unique environment to provide HIV, STI, and reproductive health education; and offer rapid HIV and syphilis testing and counseling at a testing booth. Clients who were interested in HIV and syphilis testing had to complete a pre-counseling form using a creative name that was linked to the client ID to assess risk behaviors and complete a pre-test on HIV knowledge before receiving HIV and STI education and testing. A medical technologist collected blood samples by venipuncture and performed on-site testing using rapid third-generation HIV assay (Alere Determine^TM^ HIV-1/2, Bioline HIV-1/2) and rapid syphilis assays (Alere Determine^TM^ TP). The counselor conducted post-test counseling for all clients and referred clients to receive appropriate prevention and care based on their risk behaviors and HIV status, similar to the OP flow. The counselor obtained contact detail of all participants and contacted them via phone within 30 days after on-site testing to provide additional support and ensure that participants were linked to appropriate care.

### Data collection

Our period of analysis is from September 2018 through July 2021. *For OP*, online staff collected data in the registration form and counselor case record forms. Participants who attended OP or returned to OP 2 months after the first visit were counted as a new client. For POA, the counseling staff collected data from pre-counseling form and case record forms after each event and combined data from all events between July 2018 to February 2020.

### Informed consent

No informed consent was required as OP and POA were considered by the Thailand Ministry of Public Health as routine program implementation. Results from the analysis cannot be linked to individual respondents as the online survey and data from outreach activities including linkage to ART and PrEP services data are anonymous.

### Statistical analysis

All datasets were merged, cleaned, and analyzed using STATA 15.1 (StataCorp, Lakeway Drive, College Station, Texas). We used frequency and percentage to describe categorical data, and used median, interquartile range (IQR) to describe continuous data. χ2 or Fisher’s exact test was used for comparison between groups among categorical data. The Kruskal-Wallis test by ranks (Kruskal-Wallis H test) was used to test the medians of three or more independent groups. Logistic regression was performed to explore factors associated with HIV positivity. Multiple logistic regression was used to determine independent factors. All statistic tests were performed with a significance level of 0.05.

## Results

From September 2018 to July 2021, 3,972 clients participated in the YM2M program, of which 2,973 were reached by OP and 999 by POA. For POA, 328 and 671 participants were reached at 9 events of Mor-Lam concerts and 16 events at schools/vocational colleges Bangkok, respectively (Table 1).

**Table 1:** Demographic data, HIV and syphilis testing results, and linkage to prevention and treatment services among all participants by outreach strategy, Bangkok, July 2018 –July 2021.

Characteristics	Online outreach Platform	Physical Outreach Activities			P-value^A^
		Mor-Lam	High school/College	Total	
	N = 2,973	N = 328	N = 671	N = 999	
	n (%)	n (%)	n (%)	n (%)	
Median age in years (IQR)	25.0 (21.0–30.0)	26.2 (21.0–33.8)	16.0 (14.5–18.0)	17.7 (15.5–24.0)	<0.01
Age group					
<15 years	2 (0.1)	-	184 (27.4)	184 (18.4)	<0.01
15 to <25 years	558 (18.8)	153 (46.6)	408 (60.8)	561 (56.2)	
25 to <50 years	668 (22.5)	151 (46.0)	49 (7.3)	200 (20.0)	
50 years and older	21 (0.7)	22 (6.7)	14 (2.1)	36 (3.6)	
No information	1,724 (58.0)	2 (0.6)	16 (2.4)	18 (1.8)	
Self-reported gender identity					
Bisexual	-	7 (2.1)	2 (0.3)	9 (0.9)	<0.01
Cisgender female	417 (14.0)	40 (12.2)	300 (44.7)	340 (34.0)	
Homosexual female	-	-	1 (0.1)	1 (0.1)	
Cisgender male	372 (12.5)	85 (25.9)	298 (44.4)	383 (38.3)	
Homosexual male	567 (19.1)	172 (52.4)	44 (6.6)	216 (21.6)	
Transgender woman	36 (1.2)	24 (7.3)	12 (1.8)	36 (3.6)	
No information	1,581 (53.2)	-	14 (2.1)	14 (1.4)	
Risk behavior					
Unprotected sex	68 (2.3)	166 (50.6)	426 (63.5)	592 (59.3)	<0.01
Multiple partners	5 (0.2)	113 (34.4)	48 (7.1)	161 (16.1)	<0.01
Condom break	20 (0.7)	12 (3.7)	14 (2.1)	26 (2.6)	<0.01
Sexual contact with high-risk or confirmed HIV case	86 (2.9)	3 (0.9)	1 (0.1)	4 (0.4)	<0.01
Suspected or confirmed STI	592 (19.9)	1 (0.3)	1 (0.1)	2 (0.2)	<0.01
Not specified	1,840 (61.9)	1 (0.3)	1 (0.1)	2 (0.2)	<0.01
No information	362 (12.2)	32 (9.8)	180 (26.8)		<0.01
Prior HIV testing	6 (0.2)	163 (49.7)	61 (9.1)	224 (22.4)	<0.01
Received testing					
HIV	631 (21.2)	323 (98.5)	647 (96.4)	970 (97.1)	<0.01
Syphilis	57 (1.9)	323 (98.5)	647 (96.4)	970 (97.1)	<0.01
HIV test result	n = 631	n = 323	n = 647	n = 970	
Positive	286 (45.3)	37 (11.5)	4 (0.6)	41 (4.2)	<0.01
Inconclusive	-	4 (1.2)	-	4 (0.4)	
Negative	345 (54.7)	282 (87.3)	643 (99.4)	925 (95.4)	
Syphilis test result	n = 57	n = 323	n = 647	n = 970	
Reactive	35 (61.4)	46 (14.2)	4 (0.6)	50 (5.2)	<0.01
Non-reactive	22 (38.6)	277 (85.8)	643 (99.4)	920 (94.8)	
Both test results	n = 48	n = 323	n = 647	n = 970	
HIV positive & Syphilis reactive	8 (16.7)	17 (5.3)	2 (0.3)	19 (2.0)	<0.01
HIV inconclusive & Syphilis reactive	-	2 (0.6)	-	2 (0.2)	0.19
Linked to care services among HIV positive and inconclusive	n = 286	n = 41	n = 4	n = 45	
Yes	175 (61.2)	22 (53.7)	1 (25.0)	23 (51.1)	0.08
No	64 (22.4)	12 (29.3)	-	12 (26.7)	
Unknown	47 (16.4)	7 (17.1)	3 (75.0)	10 (22.2)	
Linked to PrEP/PEP services among HIV negative	n = 345	n = 282	n = 643	n = 925	
PrEP	17 (4.9)	20 (7.1)	4 (0.6)	24 (2.6)	<0.01
PEP	43 (12.5)	-	-	-	
No PrEP or PEP	8 (2.3)	-	-	-	
Unknown	277 (80.3)	262 (92.9)	639 (99.4)	901 (97.4)	

Note: P-value

^A^ comparing between Online Outreach Platform, Physical Outreach at Mor-Lam, and Physical Outreach at high school/college.

Among 2,230 participants who reported their age, the median age was 25.0 years (IQR 21.0–30.0), 26.2 (IQR 21.0–33.8) and 16.0 (14.5–18.0) among OP, Mor-Lam and school/vocational college, respectively. Fourteen participants older than 50 years in high school/colleges were teachers or school officers. Most participants in OP did not report their gender identity while more than half of participants in Mor-Lam identified themselves as homosexual male and almost half of participants in schools/colleges identified themselves as cisgender female.

The most common self-reported risk behavior was unprotected sex among Mor-Lam and in school/college participants. Majority of POA participants tested for HIV (970/999; 97.1%) while less than a quarter of OP participants tested for HIV despite receiving virtual counseling and referral to their preferred HIV testing site (631/2,973; 21.2%). However, HIV-positive test results were significantly higher among OP participants (286/631; 45.3%) than Mor-Lam participants (37/323; 11.5%), and high school/vocational college students (4/647; 0.6%). Of HIV-positive and HIV-inconclusive participants,175/286 (61.2%) in OP and 23/45 (51.1%) in POA group were successfully linked to care for confirmatory testing before initiation of HIV care or treatment. Of HIV-negative participants, the proportion of successful linkage to PrEP and PEP services was small although noted to be significantly higher in OP than POA; 4.9% vs 2.6% for PrEP, and 12.5% vs 0% for PEP, respectively (Table 1).

For youth participants (n = 1,305), 560 participated in OP and 745 in POA. Overall, the median age among youth in OP (median 21.0 years; IQR 19.0–23.0 years) was higher than the median age among youth in POA (median 16.1 years: IQR 15.0–19.0 years). Approximately 25% of youth and 30% of adults (P<0.01) were self-identified as homosexual male; 37% of youth and 16% of adults (P<0.01) reported unprotected sex as their main risk behavior. The proportion of participants tested for HIV after counseling was higher in youth compared to adults (65.0% vs 48.3%, P<0.01), but HIV positivity was lower in youth than in adults (7.8% vs 25.7%, P<0.01). Linkage to care among PLHIV or people at higher risk of HIV who have an indeterminate result was similar between youth and adults (60.9% vs 66.4%, P = 0.63), but linkage to PrEP (5.4% vs 2.4%, P<0.01) and PEP (4.3% vs 1.3%, P<0.01) was significantly higher in adults (Table 2).

**Table 2:** Demographic and sexual behavior characteristics results comparing youth (13 to 24 years of age) and adults (25 years of age and older) by outreach strategy, Bangkok, July 2018 –July 2021.

Characteristics	OP (N = 1,249)			POA (N = 981)			Total (N = 2,230)		
	Youth	Adult	P-value^A^	Youth	Adult	P-value^B^	Youth	Adult	P-Value^C^
	n (%)	n (%)		n (%)	n (%)		n (%)	n (%)	
Receive counseling	560 (44.8)	689 (55.2)	-	745 (75.9)	236 (24.1)	-	1,305 (58.5)	925 (41.5)	-
Median age in years (IQR)	21.0 (19.0–23.0)	30.0 (27.0–37.0)	<0.01	16.1 (15.0–19.0)	34.0 (28.7–43.5)	<0.01	18.8 (16.0–21.7)	30.0 (27.0–38.0)	<0.01
Self-reported gender identity									
Bisexual	-	-	<0.01	4 (0.5)	5 (2.1)	<0.01	4 (0.3)	5 (0.5)	<0.01
Cisgender female	56 (10.0)	93 (13.5)		282 (37.8)	58 (24.6)		338 (25.9)	151 (16.3)	
Homosexual female	-	-		1 (0.1)	-		1 (0.1)	-	
Cisgender male	71 (12.7)	126 (18.3)		308 (41.3)	73 (30.9)		379 (29.0)	199 (21.5)	
Homosexual male	202 (36.1)	186 (27.0)		127 (17.0)	87 (36.9)		329 (25.2)	273 (29.5)	
Transgender woman	16 (2.9)	6 (0.9)		23 (3.1)	13 (5.5)		39 (3.0)	19 (2.0)	
No information	215 (38.4)	278 (40.3)		-	-		215 (16.5)	278 (30.0)	
Risks behavior									
Unprotected sex	23 (4.1)	16 (2.3)	0.07	460 (61.7)	131 (55.5)	0.09	483 (37.0)	147 (15.9)	<0.01
Multiple partners	2 (0.4)	1 (0.1)	0.59	98 (13.1)	61 (25.8)	<0.01	100 (7.7)	62 (6.7)	0.41
Condom break	5 (0.9)	7 (1.0)	0.82	18 (2.4)	8 (3.4)	0.42	23 (1.8)	15 (1.6)	0.80
Sexual contact with high-risk or confirmed HIV case	18 (3.2)	31 (4.5)	0.31	2 (0.3)	2 (0.8)	0.25	20 (1.5)	33 (3.6)	<0.01
Suspected or confirmed STI	108 (19.3)	140 (20.3)	0.67	1 (0.1)	1 (0.4)	0.42	109 (8.3)	141 (15.2)	<0.01
Not specified	388 (69.3)	451 (65.5)	0.16	1 (0.1)	1 (0.4)	0.42	389 (29.8)	452 (48.9)	<0.01
No information	16 (2.8)	43 (6.2)	<0.01	164 (21.8)	31 (12.4)	<0.01	180 (13.7)	74 (7.9)	<0.01
Prior HIV testing	6 (1.1)	-	<0.01	101 (13.6)	123 (52.1)	<0.01	107 (8.2)	123 (13.3)	<0.01
Received testing									
HIV	129 (23.0)	214 (31.1)	<0.01	719 (96.5)	233 (98.7)	0.08	848 (65.0)	447 (48.3)	<0.01
Syphilis	18 (3.2)	19 (2.8)	0.64	719 (96.5)	233 (98.7)	0.08	737 (56.5)	252 (27.2)	<0.01
HIV test result	n = 129	n = 214		n = 719	n = 233		n = 848	n = 447	
Positive	40 (31.0)	100 (46.7)	<0.01	26 (3.6)	15 (6.4)	0.16	66 (7.8)	115 (25.7)	<0.01
Inconclusive	-	-		3 (0.4)	1 (0.4)		3 (0.3)	1 (0.2)	
Negative	89 (69.0)	114 (53.3)		690 (96.0)	217 (93.1)		779 (91.9)	331 (74.1)	
Syphilis test result	n = 18	n = 19		n = 719	n = 233		n = 737	n = 252	
Reactive	12 (67.0)	4 (21.0)	<0.01	28 (3.9)	22 (9.4)	<0.01	40 (5.4)	26 (10.3)	<0.01
Non-reactive	6 (33.3)	15 (78.9)		691 (96.1)	211 (90.6)		697 (94.6)	226 (89.7)	
Both test results	n = 17	n = 16		n = 719	n = 233		n = 736	n = 249	
HIV positive & Syphilis reactive	4 (23.5)	2 (12.5)	0.66	11 (1.5)	8 (3.4)	0.10	15 (2.0)	10 (4.0)	0.102
HIV inconclusive & Syphilis reactive	-	-	-	1 (0.1)	1 (0.4)	0.43	1 (0.1)	1 (0.4)	0.44
Reported linked-to-care services among HIV positive and inconclusive	n = 40	n = 100		n = 29	n = 16		n = 69	n = 116	
Yes	28 (70.0)	68 (68.0)	0.57	14 (48.3)	9 (56.2)	0.92	42 (60.9)	77 (66.4)	0.63
No	9 (22.5)	18 (18.0)		8 (27.6)	4 (25.0)		17 (24.6)	22 (19.0)	
Unknown	3 (7.5)	14 (14.0)		7 (24.1)	3 (18.7)		10 (14.5)	17 (14.7)	
Reported link-to-PrEP/PEP services among HIV negative	n = 89	n = 114		n = 699	n = 231		n = 779	n = 331	
PrEP	7 (7.8)	6 (5.3)	0.29	12 (1.7)	12 (5.5)	<0.01	19 (2.4)	18 (5.4)	<0.01
PEP	10 (11.2)	15 (13.2)		-	-		10 (1.3)	15 (4.3)	
No PrEP or PEP	3 (3.4)	-		-	-		3 (0.4)	-	
Unknown	69 (77.5)	93 (81.6)		678 (98.3)	205 (94.5)		747 (95.9)	298 (90.0)	

Note: P-value^A^ comparing between youth and adult group in OP who reported age

P-value^B^ comparing between youth and adult group in POA who reported age

P-value^C^ comparing between youth and adult group in all population who reported age.

In multivariate analysis, the factors that associated with positive HIV test results in OP were youth (adjusted odd ratio (aOR), 0.37; 95% CI 0.16–0.81; P = 0.01) and suspected or confirmed STI (aOR, 15.39; 95% CI 7.17–33.03; P<0.01), while gender diversity (aOR, 8.43; 95% CI 1.94–36.62; P<0.01) and positive syphilis (aOR, 5.40; 95% CI 2.45–11.88; P<0.01) were independently associated with HIV-positivity in POA at Mor-Lam after adjusting for other factors (Tables 3 and 4). There was no factor found to be significantly associated with HIV-positivity in POA at school (Table 5).

**Table 3:** Risk factors associated with positive HIV testing among those who reported HIV test results through online platform, Bangkok, July 2018 –July 2021.

Factors	HIV negative (N = 345)	HIV positive (N = 286)	Univariate		Multivariate	
			Odds ratio (95% CI)	P-value^A^	Adjusted odds ratio	P-value^B^
	n (%)	n (%)			(95% CI)	
Age groups	n = 203	n = 140				
Youth	89 (43.8)	40 (28.6)	0.51 (0.32–0.81)	<0.01	0.37 (0.16–0.81)	0.01
Adult	114 (56.2)	100 (71.4)	1 (reference)	-	1 (reference)	-
Self-reported gender identity	n = 145	n = 147				
Gender-diverse	84 (57.9)	60 (40.8)	0.50 (0.31–0.80)	<0.01	1.48 (0.70–3.15)	0.30
Cisgender	61 (42.1)	87 (59.2)	1 (reference)	-	1 (reference)	-
Risk behavior	n = 345	n = 286				
Unprotected sex	3 (0.9)	-	-	-	-	-
Multiple partners	1 (0.3)	-	-	-	-	-
Condom break	5 (1.4)	-	-	-	-	-
Sexual contact with high-risk or confirmed HIV case	12 (3.5)	5 (1.7)	0.49 (0.17–1.42)	0.19	-	-
Suspected or confirmed STI	37 (10.7)	176 (61.5)	13.32 (8.79–20.19)	<0.01	15.39 (7.17–33.03)	<0.01
Syphilis result	n = 40	n = 8				
Reactive	19 (47.5)	8 (100.0)	-	-	-	-
Non-reactive	21 (52.5)	-	1 (reference)	-	-	-

Note: P-value^A^ of odds ratio.

P-value^B^ of adjusted odds ratio.

**Table 4:** Risk factors associated with positive HIV testing among those who reported HIV test results through physical outreach at Mor-Lam, Bangkok, July 2018 –July 2021.

Factors	HIV negative (N = 282)	HIV positive (N = 37)	Univariate		Multivariate	
			Odds ratio (95% CI)	P-value^A^	Adjusted odds ratio	P-value^B^
	n (%)	n (%)			(95% CI)	
Age groups	n = 280	n = 37				
Youth	125 (44.6)	23 (62.2)	2.04 (1.01–4.12)	0.05	1.88 (0.88–4.01)	0.104
Adult	155 (55.4)	14 (37.8)	1 (reference)	-	1 (reference)	-
Self-reported gender identity	n = 282	n = 37				
Gender-diverse	164 (58.2)	35 (94.6)	12.59 (2.97–53.38)	<0.01	8.43 (1.94–36.62)	<0.01
Cisgender	118 (41.8)	2 (5.4)	1 (reference)	-	1 (reference)	-
Risk behavior	n = 282	n = 37				
Unprotected sex	149 (52.8)	15 (40.5)	0.61 (0.30–1.22)	0.16	-	-
Multiple partners	93 (33.0)	18 (48.6)	1.92 (0.96–3.84)	0.06	-	-
Condom break	8 (2.8)	2 (5.4)	1.96 (0.40–9.59)	0.41	-	-
Sexual contact with high-risk or confirmed HIV case	3 (1.1)	-	-	-	-	-
Suspected or confirmed STI	1 (0.3)	-	-	-	-	-
Syphilis result	n = 282	n = 37				
Reactive	27 (9.6)	17 (45.9)	8.03 (3.76–17.14)	<0.01	5.40 (2.45–11.88)	<0.01
Non-reactive	255 (90.4)	20 (54.1)	1 (reference)	-	-	-

Note: P-value^A^ of odds ratio.

P-value^B^ of adjusted odds ratio.

**Table 5:** Risk factors associated with positive HIV testing among those who reported HIV test results through physical outreach at School, Bangkok, July 2018 –July 2021.

Factors	HIV negative (N = 643)	HIV positive (N = 4)	Univariate		Multivariate	
			Odds ratio (95% CI)	P-value^A^	Adjusted odds ratio	P-value^B^
	n (%)	n (%)			(95% CI)	
Age groups	n = 627	n = 4				
Youth	565 (90.1)	3 (75.0)	0.33 (0.03–3.21)	0.34	-	-
Adult	62 (9.9)	1 (25.0)	1 (reference)	-	-	-
Self-reported gender identity	n = 628	n = 4				
Gender-diverse	53 (8.4)	3 (75.0)	32.55 (3.33–318.39)	<0.01	-	-
Cisgender	575 (91.6)	1 (25.0)	1 (reference)	-	-	-
Risk behavior	n = 282	n = 37				
Unprotected sex	422 (65.6)	4 (100.0)	-	-	-	-
Multiple partners	48 (7.5)	-	-	-	-	-
Condom break	13 (2.0)	-	-	-	-	-
Sexual contact with high-risk or confirmed HIV case	1 (0.2)	-	-	-	-	-
Suspected or confirmed STI	-	-	-	-	-	-
Syphilis result	n = 643	n = 4				
Reactive	2 (0.3)	2 (50.0)	320.5 (29.0–3538.9)	<0.01	-	-
Non-reactive	641 (99.7)	2 (50.0)	1 (reference)	-	-	-

Note: P-value^A^ of odds ratio.

P-value^B^ of adjusted odds ratio.

## Discussion

In this program aimed at reaching young key populations, we found the OP was more effective than POA in reaching undiagnosed HIV positive individuals. Most participants who accessed OP reported never been tested, suggesting that this strategy was effective in reaching individuals who otherwise could not be reached previously. Among those who reported gender identity, gender-diverse individuals were reached more often in OP than POA due to the design of the pages to attract gender-diverse key population and half were youth. Although OP reached more participants, those engaged by POA strategy underwent testing more frequently. Youth were more likely to report specific risk and test for HIV than adults, though they were found to have lower HIV positivity yield. About half of those who tested HIV-positive were linked to care, but a low proportion of HIV negative individuals were linked to PrEP or PEP under both strategies.

More participants were reached using OP than POA probably due to the social media campaign component and online features (i.e., quick and easy access, confidentiality, no travel cost incurred). Current lifestyle of young people also fits with online access. A previous study showed that the rate of HIV testing increased by 16% after participants received online intervention and that 36% of participants who were never tested before were amenable to HIV testing subsequent to online intervention [12]. Anonymous online communication may allow participants to initiate and share information more comfortably and with less pressure, and for some individuals it may facilitate learning about HIV. Two other studies in 2017 showed that young MSM and TGW obtained appropriate knowledge regarding HIV testing and prevention online by successfully receiving testing and referral services at the appropriate research sites [13, 14]. However, our findings show that online access (OP) was less efficient in getting participants tested for HIV compared to POA. This could be due to the need to travel to a healthcare facility to perform the test and receive result, an additional step that needs to be taken by the participants themselves. Suggested strategies to increase uptake of HIV testing under OP include mailing or distribution of HIV self-test at specific points to participants.

A higher proportion of participants were tested for HIV under POA compared to OP probably due to the on-site testing option and on-site educational activities. The HIV and STI prevention knowledge was delivered more conveniently through activities such as games or other teaching tools provided at the site. On-site testing provides great opportunity for participants to experience the positive attitude of HIV testing and receive appropriate pre- and post-test HIV counseling. POA may be suitable for hotspot venues that are able to reach individuals who are less inclined to test at healthcare facilities. However, POA requires significantly more time and resources, and it is more susceptible to public health concerns such as a respiratory viral pandemic.

The Thai Northeastern folk concert or “Mor-Lam” is popular among young gender and sexually diverse individuals. We estimated that more than 40 Mor-Lam events were held in Bangkok and vicinity annually before the COVID-19 pandemic. Each event had more than 300 dancers, with more than half of the male dancers self-identifying as MSM or TG, and most events had more than 1,000 viewers Our findings from a previous study of 5 Mor-Lam concerts in Bangkok between December 2018 to June 2019 showed that among 196 all-aged MSM and TGW, 19% and 14% of participants had HIV and syphilis infection, respectively [15]. These results are consistent with our current findings from Mor-Lam concerts conducted between 2019 to 2020 as 14.2% of Mor-Lam participants had either HIV or syphilis infection or both. Although we can conveniently recruit groups placed at higher risk at Mor-Lam concerts. the implementation of HIV and STI education and testing at Mor-Lam concerts was challenging due to the loud noise and lack of confidential space to provide counseling [15]. Many tools and technologies such as a standard short pre-test HIV counseling video clip with headphone was used to overcome such distractions at the concert. Despite these challenges, our team found these Mor-Lam concerts to be a great opportunity to promote HIV/STI prevention and testing. We recommend that a testing booth be installed at each concert when planning future activities.

The UNAIDS Fast-Track campaign of ending of the HIV/AIDS epidemic, “95-95-95” goals in 2030 of Test-Treat-Retain of HIV, is still challenging [2, 6]. Our findings showed low coverage of prior HIV testing in all populations using either approach. The proportion of participants reporting prior HIV testing was lower than in other studies [8, 16, 17], including the UNAIDS report [1]. Previous study of 571 MSM and TGW over 18 years of age, who were reached with one of three approaches: online, mixed (online to offline) and offline, showed an HIV prevalence of 15.9%, 3.4%, and 13.0%, respectively [18]. Another study of 489 MSM and TGW who were reached with one of three approaches: private clinic-based, online pretest counseling with private clinic-based HIV testing (hybrid), and online based, the HIV prevalence was 2.2%, 1.4%, and 16.0%, respectively [13]. Our findings showed higher HIV prevalence than either study.

Similar to our findings, gender diversity has been observed to be associated with risk for HIV infection in many studies [3–5, 8, 13, 16, 17, 19, 20]. We found that among participants who were 13 to 25 years of age, gender-diverse participants had significant higher prevalence of both HIV and syphilis than heterosexual participants (22.2% vs 1.9% for HIV and 22.2% vs 3.8% for syphilis). We also found reactive syphilis test was significantly associated with HIV positivity, similar to previous studies [20, 21]. Syphilis, as well as other STI, share the same route of transmission as HIV. With a high prevalence of syphilis among MSM, both syphilis and HIV should be tested together in every case [22].

Our study found low linkage to care and prevention services in both approaches. Linkage to HIV testing and to PrEP and PEP if HIV negative and at high-risk needs to improve, particularly under the OP approach [1–3]. It is likely that the results are underestimated as the participants may not voluntarily report the care and services they received, particularly under OP where they need to go to a health facility for testing.

### Limitations

First, participants were not required to answer all questions, resulting in missing or incomplete data such as age, gender, risk behaviors or risk events, and testing results. Second, participants in OP and POA could be the same person as we did not collect identifying information to preclude them from participating in either strategy. Third, as the questionnaire asked about behavior during past 3 months, recall bias could have influenced participant’s ability to remember events. Fourth, we were unable to track if participants engaged or were linked to services beyond the 30-day follow up. Finally, COVID-19 pandemic may have affected access to testing under OP (i.e., reduction in HIV testing due to closure of health facilities, redirection of resources to COVID-related activities) and how often people gather (i.e., lock downs), leading to a decrease in risky behavior making testing unnecessary.

### Conclusion

While Online Outreach Platform seems to be more effective in reaching first-time HIV testers and identifying undiagnosed PLHIV than Physical Outreach Activities, the latter may still be important in reaching certain risk groups while delivering HIV and STI prevention education more readily, particularly in schools and colleges. Among Physical Outreach Activities strategies, Mor-Lam concerts reported the highest HIV-positive yield and the highest proportion of young key population recruited, while colleges reported fewer HIV-positive results. Gender diversity and reactive syphilis serology were also associated with HIV infection.

## Supporting information

S1 Dataset (XLSX)
